# Genome-Wide Identification of the Tify Gene Family and Their Expression Profiles in Response to Biotic and Abiotic Stresses in Tea Plants (*Camellia sinensis*)

**DOI:** 10.3390/ijms21218316

**Published:** 2020-11-05

**Authors:** Xin Zhang, Wei Ran, Jin Zhang, Meng Ye, Songbo Lin, Xiwang Li, Riffat Sultana, Xiaoling Sun

**Affiliations:** 1 Tea Research Institute, Chinese Academy of Agricultural Sciences, No. 9 South Meiling Road, Hangzhou 310008, China; xinzhang@tricaas.com (X.Z.); ranweitea@163.com (W.R.); zhangjin1369@tricaas.com (J.Z.); mengye@tricaas.com (M.Y.); linsongbo@tricaas.com (S.L.); lixiwang0392@tricaas.com (X.L.); 2 Key Laboratory of Tea Biology and Resources Utilization, Ministry of Agriculture, No. 9 South Meiling Road, Hangzhou 310008, China; 3 Department of Zoology, University of Sindh, Jamshoro 76080, Pakistan; riffat.sultana@usindh.edu.pk

**Keywords:** TIFY gene family, JA signalling pathway, *Ectropis obliqua*, *Colletotrichum camelliae*, *Camellia sinensis*

## Abstract

The TIFY family is a plant-specific gene family that is involved in regulating a variety of plant processes, including developmental and defense responses. The chromosome-level genome of the tea plant (*Camellia sinensis*) has recently been released, but a comprehensive view of the TIFY family in *C. sinensis* (the *CsTIFY* genes) is lacking. The current study performed an extensive genome-wide identification of *CsTIFY* genes. The phylogenetics, chromosome location, exon/intron structure, and conserved domains of these genes were analyzed to characterize the members of the *CsTIFY* family. The expression profiles of the *CsTIFY* genes in four organs were analyzed, and they showed different spatial expression patterns. All *CsJAZ* genes were observed to be induced by jasmonate acid (JA) and exhibited different responses to abiotic and biotic stresses. Six of seven *CsJAZ* genes (*CsJAZ1, CsJAZ2, CsJAZ3, CsJAZ4, CsJAZ7,* and *CsJAZ8)* were upregulated by mechanical wounding and infestation with the tea geometrid (*Ectropis obliqua*), while infection with tea anthracnose (*Colletotrichum camelliae*) primarily upregulated the expression levels of *CsJAZ1* and *CsJAZ10*. In addition, CsJAZs were observed to interact with CsMYC2 and AtMYC2. Therefore, the results of this study may contribute to the functional characterization of the *CsTIFY* genes, especially the members of the JAZ subfamily, as regulators of the JA-mediated defense response in tea plant.

## 1. Introduction

The TIFY family is a plant-specific gene family that was first discovered in *Arabidopsis,* and this family is named after the highly conserved TIFY domain, which was previously known as ZIM (zinc-finger protein expressed in inflorescence meristem) [1,2,3,4,5]. The TIFY domain contains approximately 36 amino acids, and the core motif TIF[F/Y]XG exhibits variant forms, with glycine being completely conserved [1]. According to the diverse domain structure, the TIFY family can be divided into four major subfamilies, including ZML (ZIM/ZIM-like), PPD (PEAPOD), JAZ (jasmonate–ZIM-domain) and TIFY [6]. In addition to the TIFY subfamily, which only contains the TIFY domain, the other three subfamilies also have specific and conserved domains aside from the TIFY domain. ZML subfamily proteins have a C2C2-GATA zinc finger domain responding for DNA binding and a CCT (CONSTANS/CO-like/TOC1) domain involved in protein–protein interaction. The JAZ subfamily contains another conserved sequence, the Jas motif (CCT_2 domain), which is similar to the N-terminal sequence of the CCT domain and is characterized by SLX_2_FX_2_KRX_2_RX_5_PY in the C-terminus. The PPD protein has a unique PPD domain in the N-terminus and a truncated Jas domain that lacks the conserved PY residue [6,7,8]. In addition, the TIFY family can also be classified into two groups depending on the presence (Group I) or absence (Group II) of the C2C2-GATA domain. The ZML subfamily belongs to Group I, while the JAZ, PPD, and TIFY subfamilies belong to Group II [9,10].

The TIFY family has recently been analyzed in several sequenced plant genomes, including those of *Arabidopsis* (*Arabidopsis thaliana*), rice (*Oryza sativa*), tomato (*Solanum lycopersicum*), bamboo (*Phyllostachys edulis*), wild soybean (*Glycine soja*), and purple false brome (*Brachypodium distachyon*) [7,9,10,11,12,13]. Functional information regarding various TIFY proteins has also been obtained, demonstrating their regulatory role in the process of plant development and their responses to biotic and abiotic stresses [2,14,15,16]. ZIM and its homologous proteins ZML1 and ZML2 are GATA transcription factors that are involved in the transcriptional regulation of developmental processes. The overexpression of *AtZIM/TIFY1* in *Arabidopsis* resulted in elongated petioles and hypocotyls due to increased cell elongation independent of gibberellin and brassinosteroids [2]. Recently, it was observed that ZML2 in maize acts as a transcriptional repressor in lignin biosynthesis [15]. AtPPD1 and AtPPD2 were reported to affect leaf shape, silique length modifications, and meristemoid division in *Arabidopsis* [14], and the *PPD* orthologue *BIG SEEDS1* in leguminous plants was observed to regulate primary cell proliferation [16]. TIFY8 is a TIFY subfamily member that is primarily observed in dicotyledons and has been shown to exist in the form of a single gene, even in *Arabidopsis* [6]. TIFY8 usually acts as a possible transcriptional repressor by interacting with transcriptional regulators involved in the process of plant growth and development [17].

In all the plant species studied, JAZ proteins are the most extensively investigated TIFY subfamily because they are important components of the jasmonate acid (JA) signalling pathway, acting both as coreceptors together with the COI1 receptor and as repressors of downstream transcription factors (TFs) [3,5,18]. In the absence of JA, JAZ proteins interact with multifamily TFs, such as basic-helix-loop-helix (bHLH) protein MYC2, which regulates the expression of JA-responsive genes and acts as a repressor by simultaneously binding to the adaptor protein NINJA and the general corepressor TOPLESS. In the presence of JA, JAZs act as coreceptors with COI1 and are recruited to the SCF^COI1^ complex to perceive JA-Ile; subsequently, JAZs are degraded via 26S proteasomes, releasing the activity of TFs and enabling them to interact with other cofactors, such as MED25, to regulate JA-responsive genes [19,20,21]. JAZ proteins function as the most diverse module and interact with different proteins or transcription factors, enabling the JA signaling cascade to link various environmental inputs to diverse physiological outputs [22,23]. For example, a higher-order mutant (*jaz decuple*, *jazD*) that is defective in 10 *JAZ* genes (*JAZ1-7*, *9-10*, and *-13*) exhibited resistance to insect feeding and infection by necrotrophic fungal pathogen *Botrytis cinerea* [24]. The silencing of *NaJAZh* alone is able to alter the accumulation of two herbivore-induced defense metabolites and strongly suppress the performance of *Manduca sexta* larvae on *Nicotiana tabacum* [25]. The overexpression of *OsTIFY11a/OsJAZ9* was associated with strongly increased tolerance to salt and dehydration stresses in rice [26].

The tea plant *Camellia sinensis* (L.) O. Kuntze is an economically valuable woody plant, and this evergreen crop is continuously challenged by a wide variety of biotic stresses, such as the leaf-feeding pest *Ectropis obliqua* and the dominant leaf fungi bacterial *Colletotrichum camelliae* [27,28,29]. Previous studies based on transcriptomic strategies indicated that JAZ subfamily genes were involved in the interactions between the tea plant and biotic stresses [30]. A recent study also reported that 12 *CsJAZ* genes were identified in an older version of the tea genome [31]. However, with the recently released chromosome-level genomic sequence of the tea plant [32], information regarding the TIFY family, including the ZML, TIFY, and PPD subfamilies, is still lacking. In addition, how *CsTIFY* genes respond to biotic or abiotic stresses has not been fully elucidated. In the present study, we searched the genome database and identified 16 genes encoding TIFY proteins. Phylogenetics, chromosome location, exon/intron structure, and conserved protein domains were analyzed to characterize the members of the *CsTIFY* gene family. Moreover, the expression profiles of the *CsTIFY* genes in different organs and their responses to the exogenous application of JA, mechanical wounding, infestation by *E. obliqua*, and infection by *C. camelliae* were analyzed. Furthermore, the interactions between key CsJAZ proteins and CsMYC2 were investigated as well. Our results may facilitate the functional characterization of *CsTIFY* genes in tea plants.

## 2. Results

### 2.1. Identification of the Members of the TIFY Gene Family in the Tea Plant

**Figure 1:**
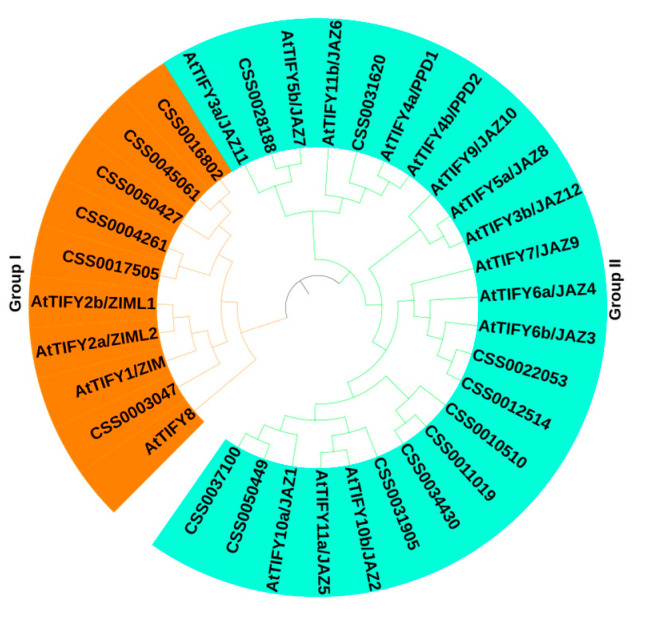
Phylogenetic tree of TIFY proteins from tea plant and Arabidopsis. The predicted full-length amino acid sequences of 16 CsTIFY and 18 AtTIFY were used to construct a phylogenetic tree using MEGA5.1 by the neighbor-joining method. The branch of Group I is marked in orange and that of Group II is marked in green.

To identify all the putative TIFY domain-containing proteins in the tea plant, an hidden Markov model (HMM) profile of the TIFY domain (PF06200) was employed to perform a blast search against the chromosome-level genome of the tea plant (downloaded from TPIA). Through this research, 16 candidate loci encoding putative TIFY proteins were identified in the tea plant. Identification within the predicted protein sequences of the tea plant genome was conducted with the keyword “TIFY”, and nine proteins were identified. These nine proteins were included in the proteins identified through the HMM file. The 16 proteins were analyzed for conserved domains in the Pfam database to reconfirm that they belong to the TIFY family. All 16 proteins were found to contain the conserved TIFY motif, and the characteristics of the TIFY motif of all CsTIFY proteins are presented in Table 1. In addition to the TIFY domain, three other domains, the CCT domain (PF06203), GATA domain (PF00320), and Jas domain (PF09425), were also identified in the CsTIFY family. All 16 TIFY family members in tea plants were named according to the existing numbering system described in *Arabidopsis* and the phylogenetic analysis with AtTIFY and AtJAZ proteins described below (Figure 1 and Supplementary Figure S1). Members of the JAZ and PPD subfamilies have a consensus sequence, “TIFYXG”, in the TIFY motif, while most ZML proteins possess a conserved “TLSFXG” motif in the same domain. The coding sequences (CDSs) of the *CsTIFY* genes ranged from 411 bp (*CsJAZ7*) to 1197 bp (*CsJAZ3* and *CsJAZ4*) with predicted proteins of 136 to 398 amino acids.

### 2.2. Phylogenetic Analysis of the TIFY Family

To identify groups of orthologous proteins and explore the evolutionary relationship of CsTIFY, full-length sequences of 16 CsTIFY proteins, along with 18 TIFY proteins from *Arabidopsis,* were used to build a phylogenetic tree with the maximum-likelihood (ML) method (Figure 1). All 34 TIFYs were classified into two major groups (Group I and Group II). Among these proteins, proteins containing the GATA domain, including six CsZML proteins and three ZML members from *Arabidopsis,* were clustered in Group I. AtTIFY8 was clustered in Group I but not in the ZML branch, and no AtTIFY8 homologs were found among CsTIFY in this analysis. Group II contains proteins with the absence of the GATA domain except for CsZML7 and CsZML8, including all the members of the JAZ and PPD subfamilies from *Arabidopsis* and putative homologues in tea plant.

**Figure 2:**
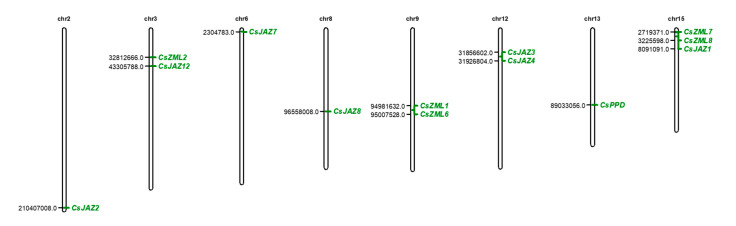
Chromosomal location of CsTIFY genes. A total of 13 CsTIFY genes were mapped to the eight chromosomes according to their positions in the tea plant genome. The chromosome number was shown on the top of each chromosome.

Gene duplication is an evolutionary event that contributes to the multimember gene family. Thus, to characterize the distribution of CsTIFY family genes, the chromosomal locations of all 16 *CsTIFY* genes were determined based on the pseudochromosome assembly of the tea plant genome. The 13 *CsTIFY* genes were mapped on eight chromosomes, including chromosomes 2, 3, 6, 8, 9, 12, 13, and 15, with 3 *CsTIFY* genes being mapped to chromosome contigs (Figure 2). *CsTIFY* genes were generally dispersed in the tea plant genomes, with no more than three *TIFY* genes being present on one chromosome. In addition, *CsZML7*, *CsZML8, CsJAZ3*, and *CsJAZ4*, which are homologous genes, are adjacent to each other in the same chromosome; *CsZML2* and *CsZML3* are also segmental duplication genes, although they were mapped to different contigs or chromosomes.

### 2.3. Sequence Analysis of the CsTIFY Family

**Figure 3:**
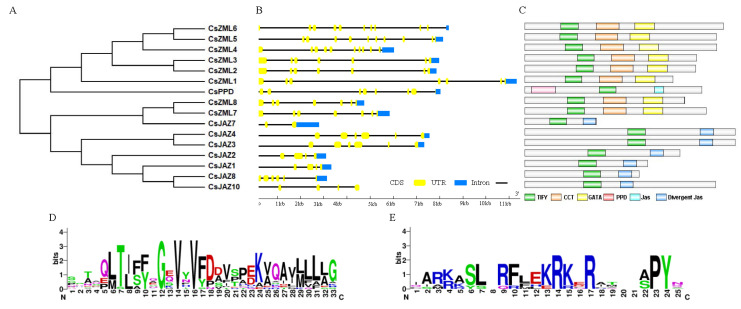
Sequence analysis of TIFY in tea plant. (**A**) Phylogenetic analysis of CsTIFY proteins. The phylogenetic tree was performed in MEGA5.1 software with the neighbor-joining method. (**B**) Exon/intron structure of CsTIFY genes. Exons and introns were represented by yellow boxes and black lines, respectively. (**C**) The distribution of conserved domains in CsTIFY proteins. Each domain was represented by a colored box. Sequence logos of the TIFY (**D**) and Jas (**E**) domains from CsTIFY proteins.

To obtain more diversified information within the CsTIFY family, a phylogenetic tree, exon–intron organization, and conserved domains were constructed within the *CsTIFY* gene family (Figure 3). The results showed that 16 TIFY proteins were clearly divided into three subgroups: the JAZ subfamily, ZML subfamily, and PPD subfamily, with the exception of CsZML7 and CsZML8 (Figure 3A). The exon–intron organization within the *CsTIFY* gene family indicated that all the gene members processed introns, and the number of introns was variable (Figure 3B). Although the gene structures of the three subfamilies are clearly different from one another, most genes in the same subfamily tend to have the same member of introns and the same splicing positions, such as *CsJAZ1*, *CsJAZ2* and *CsZML1*, *CsZML2*. Conserved domains in CsTIFY were analyzed in the Pfam web server (Figure 3C). In addition to the TIFY domain, seven members of the CsJAZ subfamily also contain a Jas/CCT_2 domain (PF09425), CCT domain (PF06203), and GATA domain (PF00320) and were also found in eight CsZML subfamily proteins. A PPD domain and modified Jas domain were found in one CsPPD. The protein domain architecture was mostly in accord with the clustering mode of the phylogenetic analysis. CsZML7 and CsZML8 were not included in the ZML branch, but they have the same gene and domain structure as other ZML members.

Furthermore, the conservation of functional motifs in the TIFY domain among CsTIFY proteins, as well as the Jas motif among CsJAZ proteins, was also performed by drawing their sequence logos. The results showed that the entire TIFY domain showed diverse forms, but most of them are conserved within the TIF[F/Y]XG region and amino acid sites Val-14 and Val-16, as presented in Figure 3D. Among these motifs, the TIFY motifs in the JAZ and PPD subfamilies exhibited conserved TIFYXG, while those of the ZML subfamily deviated from TIF[F/Y]XG, including TLSFXG (five proteins), TLAFEG (two proteins), and TSELSI. Jas motifs are highly conserved, particularly in location sites 6, 7, 9, 10, 14, 17, 23, and 24 (Figure 3E). In addition, the amino acid PY located at the C-terminus of the Jas motif is entirely conserved, and it is a symbol used to distinguish the Jas motif in the JAZ subfamily from the divergent Jas motif in the PPD subfamily.

### 2.4. Expression Profile of CsTIFY Genes in Different Organs

**Figure 4:**
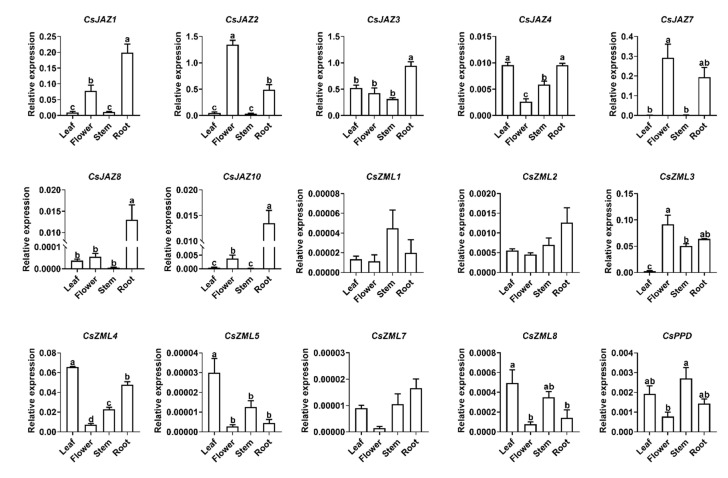
Expression profile of *CsTIFY* genes in four organs. Data are shown by the mean + SE, different letters indicate significant difference among different organs (Turkey’s honest significant difference (HSD) post-hoc test, *p* < 0.05, *n* = 6).

The expression patterns of *CsTIFY* genes in four main organs (leaf, flower, stem, and root) were investigated using qRT-PCR (Figure 4). Among the 16 *TIFY* genes, 15 were expressed in at least one organ, except *CsZML6*, whose amplification failed because of its low expression level in those detected organs. *CsJAZ4*, *CsZML4, CsZML5,* and *CsZML8* showed their greatest relative abundance in leaves; *CsJAZ2*, *CsJAZ7,* and *CsZML3* were expressed at higher levels in flowers than in the other organs; *CsJAZ1, CsJAZ3, CsJAZ8,* and *CsJAZ10* had a stronger expression level in roots than in the other organs. The expression levels of *CsZML1*, *CsZML2*, and *CsZML7* in the four organs were similar and exhibited no significant difference. The transcription levels of the only member of the PPD subfamily *CsPPD* in stems and leaves were higher than those in flowers and roots. Overall, the relative expression levels of the 15 *CsTIFY* genes in four organs exhibited distinct expression patterns.

### 2.5. Expression Profiles of CsTIFY Genes under JA and Mechanical Wounding Treatment

**Figure 5:**
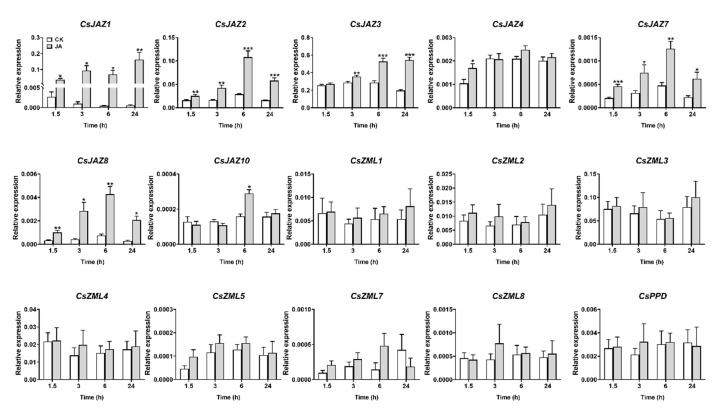
Expression profile of *CsTIFY* genes elicited by exegenous application of jasmonate acid (JA). Data are shown by the mean + SE, asterisks indicate a significant difference between treatment and control (Student’s *t*-test, * *p* < 0.05, ** *p* < 0.01, *** *p* < 0.001, *n* = 6).

**Figure 6:**
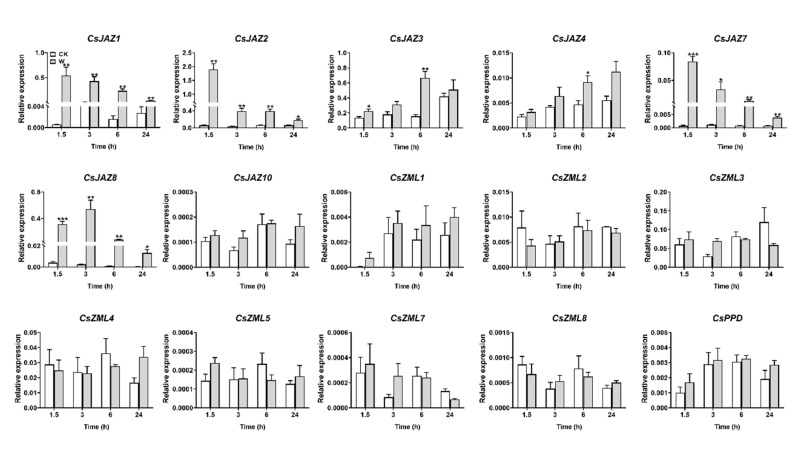
Expression profile of *CsTIFY* genes elicited by mechanical wounding. Data are shown by the mean + SE, asterisks indicate significant difference between treatment and control (Student’s *t*-test, * *p* < 0.05, ** *p* < 0.01, *** *p* < 0.001, *n* = 6).

We first investigated the transcript levels of *CsTIFY* genes in leaves treated with an exogenous application of JA and mechanical wounding (Figure 5, Figure 6). The transcription levels of most genes in the JAZ subfamily were significantly increased in response to JA and mechanical wounding. After JA treatment, *CsJAZ1*, *CsJAZ2*, *CsJAZ7,* and *CsJAZ8* were induced more than tenfold at most time points from 1.5 to 24 h, and the fold change for *CsJAZ3* and *CsJAZ4* was approximately fivefold for at least one time point, while *CsJAZ10* was slightly induced at 6 h (Figure 5). The expression of *CsJAZ1*, *CsJAZ2*, *CsJAZ7,* and *CsJAZ8* was also strongly upregulated within 1.5 h of mechanical wounding, with transcription levels gradually decreasing over time (Figure 6). In contrast, the accumulation of *CsJAZ3* and *CsJAZ4* induced by mechanical wounding was delayed and peaked at 6 h, while mechanical wounding treatment did not induce the expression of *CsJAZ10*. Other *CsTIFY* genes in the ZML and PPD subfamilies showed no significant change in expression level after those two treatments.

### 2.6. Expression Profiles of CsTIFY Genes in Response to Attack by the Tea Geometrid and Tea Anthracnose

**Figure 7:**
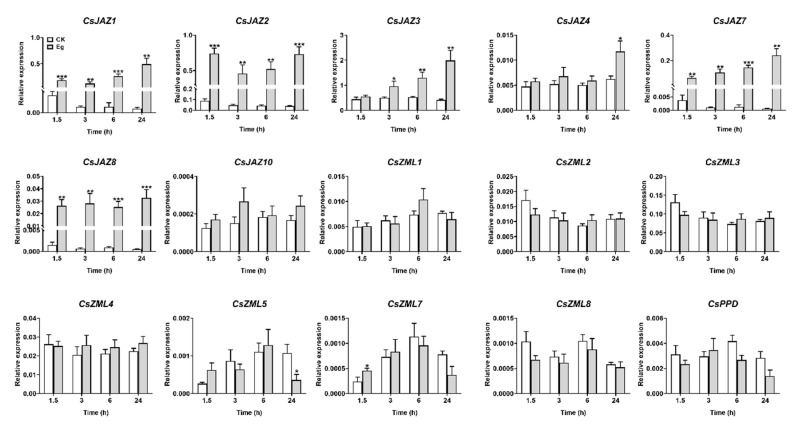
Expression profile of *CsTIFY* genes elicited by the infestation of *E. obliqua*. Data are shown by the mean + SE, asterisks indicate significant difference between treatment and control (Student’s *t*-test, * *p* < 0.05, ** *p* < 0.01, *** *p* < 0.001, *n* = 6).

**Figure 8:**
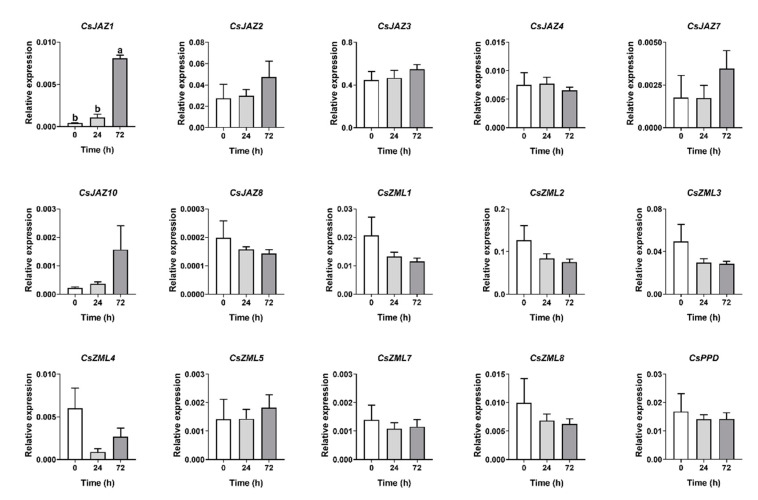
Expression profile of *CsTIFY* genes elicited by *C. camelliae* infection. Data are shown by the mean + SE, different letters indicate significant difference among different organs (Turkey’s honest significant difference (HSD) post-hoc test, *p* < 0.05, *n* = 6).

Members of the JAZ family exhibited different expression patterns in response to different biotic stresses in many plant species, leading us to investigate how members of the *CsJAZ* subfamily respond to attacks by insect herbivores and pathogens. The results showed that six members of the *CsJAZ* subfamily were upregulated by damage by tea geometrid with different response patterns, as well as *CsJAZ10* (Figure 7). Geometrid attack significantly upregulated the expression levels of *CsJAZ1*, *CsJAZ*7, and *CsJAZ*8 at least 15-fold at all tested time points, and *CsJAZ*2 was also induced at least eightfold at four time points. Transcription levels of *CsJAZ3* increased at 3 h, 6 h, and 24 h, and *CsJAZ4* only increased at 24 h. On the other hand, *CsJAZ1* and *CsJAZ10* were the two genes that were upregulated after 72 h of infection with tea anthracnose (Figure 8), suggesting that the induction of these two genes was primarily responsive to tea anthracnose. Other *CsTIFY* genes in the ZML and PPD subfamilies were not responsive to those two biotic stresses except for *CsZML5*, which was slightly downregulated, and *CsZML7*, which was slightly upregulated by geometrid attack.

### 2.7. Interactions of CsJAZ with CsMYC2

**Figure 9:**
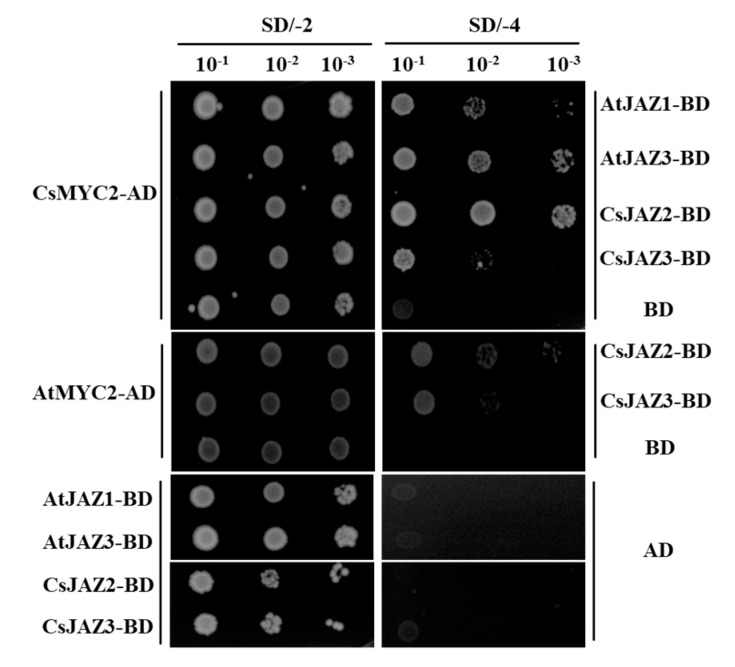
CsJAZs interacted with CsMYC2 and AtMYC2 in yeast two-hybrid assay. The AtJAZ1-BD and AtJAZ3-BD were used as positive control, while empty vector pGBKT7 plus CsMYC2-AD/ AtMYC2-ADand pGADT7 plus CsJAZ-BD/ AtJAZ-BD were set as negative control. The yeast cells were grown on SD/-Trp-Leu and SD/-Trp-Leu-His-Ade media with three concentration gradients.

Interaction with MYC2 is a general feature of JAZ repressors in other plants. We selected CsJAZ2 and CsJAZ3, which were highly expressed and exhibited different responses to JA, to assess their capacity of interacting with CsMYC2 and AtMYC2 using yeast two-hybrid experiments. As the results of this study showed that CsJAZ2 and CsJAZ3 interacted with both CsMYC2 and AtMYC2, indicating that CsJAZ may also function in the tea plant by interacting with MYC2 (Figure 9).

## 3. Discussion

The TIFY family is an important plant-specific gene family and has been identified in multiple species among both monocots and eudicots [1,8,10,12]. Continuing research has demonstrated that the members of the TIFY gene family, especially members of the JAZ subfamily, play multiple roles in different plant developmental processes and the process of defense against biotic and abiotic stresses [1,8,10,11,13,31]. As evergreen plants, tea plants are frequently challenged by abiotic and biotic stresses, but information on the TIFY gene family is lacking, which hampers the research investigating resistance mechanisms [27,28,31]. The chromosome-level genomic sequence of the tea plant has been recently released, and the extensive annotated information provided in the tea genome provides fundamental material for comprehensive analysis of the CsTIFY family [32]. All the above-mentioned research enables us to study the evolutionary history and expression diversity of the TIFY family in tea plants.

In the present study, 16 CsTIFY proteins were identified from the chromosome-level tea genome sequence through conserved sequence analysis of the TIFY motif (Table 1). According to the reported plants, members of the TIFY family vary from one in *Marchantia polymorpha* to 48 in the higher plant *Panicum virgatum* [6,8]. TIFY in the tea plant ranks next to last with 16 members, further indicating functional diversity and redundancy in this rapidly evolving gene family. The TIFY family in tea plant was also divided into two groups, and eight CsZML proteins in Group I were believed to act as transcription factors [2]. Proteins in Group II contain JAZ repressors and PPD proteins that lack the GATA domain. This result was similar to the findings of the phylogenetic analysis of TIFYs from *Arabidopsis* and rice, further suggesting functional conservation in different plant taxa, and both groups appeared before divergence in monocots and dicots [1]. However, only three subfamilies of TIFY were found in the tea plant, including seven belonging to the JAZ subfamily, eight belonging to the ZML subfamily, and one belonging to the PPD subfamily without the TIFY subfamily. It was confirmed that not all subfamilies were found in plant species, and the TIFY subfamily is also absent from *B. distachyon* and *Sorghum bicolor* [6,13], while the PPD subfamily only exists in the dicots and in lycophyte *Selaginella moellendorffii* [6]. In addition, orthologues of AtTIFY8, which was the first member of the TIFY subfamily, are also mostly present as unique genes within the species studied [17]. One reason for the absence of the TIFY subfamily in the tea plant is that instead of TIFY subfamily genes, other genes have evolved to function as *AtTIFY8* [10]. The other reason for this exception may be the incomplete genomic sequencing of tea plants, such as that found in *indica* rice [10].

Analysis of chromosome position, intron/exon structures, and conserved domains was also employed to obtain evolutionary clues within the CsTIFY family. Segmental duplications were found in the CsTIFY family, mainly in the CsZML and CsJAZ subfamilies. Our results agree with the duplication phenomenon identified within the TIFY family, supporting the notion that duplication events play an important role in the evolution and expansion of the TIFY family in plants [6,7,8,33]. CsTIFY members in the same subfamily generally possessed a similar gene structure and shared the same distribution of conserved domain patterns, indicating the strong correlation between phylogeny and intron/exon diversification and conserved domain patterns within CsTIFY. The correlation is consistent with findings in *Arabidopsis* and other plants, which played an important role in the evolution of multiple gene families [7,33]. Among these genes, the three sets of homologous genes (*CsZML2* and *CsZML3*, *CsZML7* and *CsZML8*, *CsJAZ3* and *CsJAZ4*) contained the exact same intron/exon structures and conserved domains but with different genomic lengths, respectively, further indicating that these TIFY genes may be the products of duplication events. Furthermore, sequence logos of the TIFY domain showed that this central typical motif could be detected with slight divergence in all CsTIFY proteins except for CsZML5, sufficiently suggesting broad conservation of the main motifs characteristic of the plant TIFY family during evolution. However, the JAZ subfamily contains the same core motif as “TIFYXG”, and CsPPD also contains the “TIFYXG” motif, while it is “TLXFXG” in the ZML subfamily. The CsPPD subfamily, which has the same TIFY motif, was also clustered with CsJAZs together in both polygenetic trees, confirming that PPD proteins may be the evolutionary precursors of JAZ and may have evolved as two independent subfamilies by gene duplication in vascular plants [1,6]. A recent study reported that 12 CsJAZ proteins were found through TIFY and Jas domain blast in tea plants [31]. Among the 12 CsJAZ proteins, six members were identical to those in our study, and the other six CsJAZ proteins in the above study both contain the so-called Jas domain, which was only part of the CCT domain but was not a true Jas domain. In addition, the GATA domain was also found in their protein sequence; therefore, they should be named ZML proteins, rather than JAZ proteins [1,6,8]. In addition, the Jas motif defines JAZ proteins and mediates complex formation through direct interactions between JAZs and TFs (such as MYC2) [34,35,36]. This motif is also conserved among CsJAZ proteins, and interactions between CsJAZ2, CsJAZ3, and CsMYC2 were also suggested that CsJAZ proteins may act as functional repressors of CsMYC2 in tea plants.

All seven *CsJAZ* genes found in our study were upregulated by JA. The phylogenetic tree showed that the stronger inducible genes *CsJAZ1*, *CsJAZ2, CsJAZ7*, and *CsJAZ8* were grouped closer than *CsJAZ3* and *CsJAZ4*, which may suggest that JA-induced *CsTIFY* genes evolved from common ancestors responsive to JA [7,37]. The identical *CsJAZ* genes found in a previous study showed that *CsJAZ2, CsJAZ3,* and *CsJAZ4* were remarkably upregulated by MeJA [31]. This difference may be attributable to such factors as different tea varieties, discrepancies in the elicitation effects of JA and MeJA, and differing treatment concentrations. However, the expression level of gene members from *CsZML* and *CsPPD* was not modulated by JA in the current study. Due to the key role of JA and JAZ in plant responses to biotic and abiotic stresses, our study mainly focused on the influence of biotic and abiotic stresses on the CsJAZ subfamily. Except for *CsJAZ10*, six other *CsJAZs* were significantly upregulated after mechanical wounding, and the transcripts of most genes accumulated to higher levels within 1.5 h. Mechanical damage results in rapid accumulation of JA within 30 min and thus reshapes the expression profile of genes related to the JA signaling pathway [37]. The expression pattern of *CsJAZ* genes in response to *E. obliqua* infestation is similar to those elicited by mechanical wounding, for the *CsJAZ* members induced by mechanical wounding were also induced by *E. obliqua* feeding. However, there was also a notable difference in the induction trend for those genes. The elicitation of all *CsJAZ* by tea geometrid feeding showed a longer duration, while an induction by mechanical wounding gradually declined at later time points. This finding may be observed because mechanical wounding was not a continuous damage as feeding by tea geometrid, or herbivore-associated molecular patterns from *E. obliqua* may elongate the elicitation effect, which needs to be clarified in future studies. Previous findings also established the role of JAZ proteins in the regulation of plant anti-insect defense [25,36,37]. Several *JAZ* genes were proven to be responsive to mechanical wounding and the damage of *E. obliqua* in a previous study based on transcriptomic analysis [30]. Feeding by generalist *Spodoptera exigua* increased the transcript levels of most *AtJAZ*, while transgenic line *AtJAZ1∆3A* plants that overexpressed a Jas-motif-deleted form (AtJAZ1∆3A) perturbed JA signaling and decreased plant resistance to insect feeding [37]. The herbivory-elicited gene *NaJAZh* was proven to regulate part of JA-dependent direct and indirect defenses in tobacco, such as the accumulation of nicotine and the emission of volatile organic compounds [25]. The key roles played by JA as signals mediating pathogen defense responses have been widely documented [38]. Changes in the transcription levels of seven *CsJAZ* genes after fungal pathogen infection exhibited different expression patterns. In our assay, *CsJAZ1* and *CsJAZ10* were primarily induced by inoculation with the pathogen *C. camelliae,* providing evidence to support the hypothesis that at least two members of the CsJAZ subfamily are involved in the plant pathogen resistance of tea plants. Eight of the 12 *JAZ* genes in *Arabidopsis* were induced after infection with the bacterial pathogen *P. syringae* strain DC3000, and *AtJAZ10* was proven to be involved in the branch of JA-mediated DC3000 symptom development [7,39]. Moreover, *CsJAZ2* and *CsJAZ3* were also regulated by other hormone treatments, including ETH (ethylene), GA (gibberellin), and ABA (abscisic acid), and abiotic stress drought (PEG) [31]. Thus, although both responses are mediated by JA signaling, there are also unique regulatory events governing the expression of specific JAZ genes in response to different stimuli. Furthermore, the interaction between CsJAZ and CsMYC2 may suggest that CsJAZ participates in signal transduction and gene regulation in the defense response of tea plants by interacting with CsMYC2 to regulate the JA signaling pathway. Taken together, these results indicate that different *CsJAZ* genes were differentially responsive to various stresses, which is another possible way to provide the mechanism specificity underlying JA responses.

In conclusion, a total of 16 *TIFY* genes were identified through genome-wide analysis, and most of these genes may share common ancestors with those in other reported species, such as *A. thaliana*. Expression profile analyses showed that *CsJAZ* genes were induced by JA, mechanical wounding treatment, and biotic stresses, such as herbivorous attack and pathogen infection. Among these genes, *CsJAZ1, CsJAZ2*, *CsJAZ3, CsJAZ7,* and *CsJAZ8* were responsive to tea geometrid infestation, while tea anthracnose infection mainly regulated the expression of *CsJAZ1* and *CsJAZ10*. In addition, the interaction complex of CsJAZ–CsMYC2 also indicated their regulatory role in the JA-mediated defense response. Our results would be helpful in laying the foundation for the further functional characterization of Cs*TIFY* genes. Further investigations are also warranted to determine the function of these JAZ genes involved in the tea plant defense response.

## 4. Materials and Methods

### 4.1. Insect and Fungi Pathogen

The *E. obliqua* larvae and *C. camelliae* were originally obtained from the tea plantation of Tea Research Institute of the Chinese Academy of Agricultural Sciences (TRICAAS), Hangzhou, China. *E. obliqua* larvae was reared with fresh tea shoots of *Longjing 43*. *C. camelliae* was cultured on potato dextrose agar (PDA). The larvae and pathogen were kept in an incubator at 26 ± 2 °C, 12/12 h photo phase, and after one generation, they were used for the experiments. 

### 4.2. Plant Materials and Treatments

Two-year-old *Longjing 43* tea plants were planted individually in plastic pots (14 cm diameter × 15 cm high) and grown in a greenhouse (26 ± 2 °C, 12/12 h photo phase, 65 ± 5% room humidity). They were irrigated (once every other day) and fertilized (once a month) with the same standards. Plants without disease and insects were used for the experiments. 

#### 4.2.1. Different Organs

Various organ samples, including flowers, leaves, stems, and roots, were harvested and frozen in liquid nitrogen; then, they were stored at −80°C. Six replications were carried out.

#### 4.2.2. JA Treatment

JA (Sigma Chemical Co., St. Louis, MO, USA) was dissolved in a small amount of ethanol and then made up to 150 μg/mL in 50 mM sodium phosphate buffer (titrated with 1 M citric acid until pH 8). Plants were individually sprayed with 8 mL of JA solution. Control plants were sprayed with buffer with a small amount of ethanol. Samples were harvested at 1.5, 3, 6, and 24 h after the start of treatment, separately. Six replications were carried out.

#### 4.2.3. Mechanical Wounding Treatment

The mechanical wounding damage was made through a fabric pattern wheel for rolling with 4 times on each leaf. Six replications were carried out.

#### 4.2.4. *E. obliqua* Treatment

The second leaves of tea plants were covered with a fine-mesh sleeve and introduced with 10 2nd-instar caterpillars that had been starved for 8 h. Leaves covered with a fine-mesh sleeve but without insects were set as control. Control and insect-treated leaves were harvested at 1.5, 3, 6, and 24 h after the start of treatment, separately. Six replications were carried out.

#### 4.2.5. *C. camelliae* Infection

*C. camelliae* has been cultured on potato dextrose agar for 7 days. The plant leaves were damaged with a sterile needle 5 times, and fungus cake (ID = 6 mm) was applied to the damaged part immediately (*n* = 12). After inoculation, the potted tea plants were maintained in the greenhouse, in which conditions were 26 ± 2 °C, 90 ± 5% RH (relative humidity), and 12 h photoperiod. Then, 24 and 72 h later, the inoculated leaves were harvested and frozen in liquid nitrogen, after which they were stored at −80°C. Six replications were carried out.

### 4.3. Identification of TIFY Family in Tea 

Genome-wide screen of putative TIFY proteins in tea plant was performed within the Tea Plant Genome Database (http://tpia.teaplant.org/download.html) with a hidden Markov model (HMM) profile of the TIFY domain (PF06200) as a query and set as E-value < e^−6^. TIFY was also used as a keyword for searching protein sequences of the chromosome-level genome of tea plant. 

### 4.4. Bioinformatics Analysis of TIFY Family in Tea 

Sequence information of putative TIFYs in tea plants was also extracted from the Tea Plant Genome Database. The Pfam database (http://pfam.xfam.org/) was used for analyzing the protein domain of the TIFY proteins family. The *CsTIFY* genes were positioned on the tea chromosomes based on the genomic sequences of these genes in the Pseudochromosome assembly of the tea plant genome available at http://tpia.teaplant.org/download.html. Mapchart software was used to draw the Chromosome position (https://www.wur.nl/en/show/Mapchart.htm). An exon/intron structure schematic diagram was performed with Gene Structure Display Server (http://gsds.cbi.pku.edu.cn/). WebLogo (http://weblogo.berkeley.edu/logo.cgi) was used to draw a sequence logo of the TIFY and Jas domain. The phylogenetic trees were constructed with MEGA 5.0 using the neighbor-joining (NJ) method and a bootstrap test 1000 times.

### 4.5. RNA Extraction and qRT-PCR Anlaysis

All the samples were finely ground in liquid nitrogen. Total RNA was extracted with the RNAprep pure Plant Kit (Tiangen, Beijing, China) according to the manufacturer’s instructions. Then, 1 μg total RNA were used for cDNA preparation using PrimeScript™ RT Master Mix (TAKARA, Dalian, China) according to the manufacturer’s instructions, respectively. The cDNA samples were quantified using the SYBR Green kit (TAKARA, Dalian, China) with a cycler apparatus (Roche Diagnostics, Mannheim, Germany) as the manufacturer’s instructions indicated. Expression levels of target genes were normalized to *CsGADPH*. Primers used for qRT-PCR are listed in Supplemental Table S1.

### 4.6. Yeast Two-Hybrid Assays

Full-length sequences of CsJAZ2, CsJAZ3, AtJAZ1, AtJAZ3 and CsMYC2 were amplified with the primers described in supplementary Table S1. PCR products of CsJAZ2, CsJAZ3 digested with EcoRI and BamHI, and AtJAZ1, AtJAZ3 digested with EcoRI and SalI, were cloned into EcoRI-BamHI or EcoRI-SalI digested pGBKT7 (Clontech) separately. PCR products of CsMYC2 digested with NdeI and SmaI were cloned into the NdeI-SmaI digested plasmid pGADT7 (Clontech). The empty vectors pGBKT7 and pGADT7-CsMYC2, pGADT7 and pGBKT7-CsJAZ2/CsJAZ3/AtJAZ1/AtJAZ3, were co-transformed as negative controls. All the constructs were sequence verified, and positive plasmids were transformed into yeast (*Saccharomyces cerevisiae*) strain AH109 cells. Successful transformation in yeast cells was confirmed by solid synthetic defined (SD) media lacking Leu and Trp (SD/-2). Transformed colonies identified on SD/-2 were resuspended in liquid SD/-2 media for 6 h, and the cell density was adjusted to OD600 ≈ 1. Then, 5 μL cell suspensions were spread on SD/-2 (lacking Leu and Trp) and SD/-4 (lacking Ade, His, Leu, and Trp) to test protein interaction. Plates were incubated at 28 °C for 3 to 4 d to observe the interactions. 

### 4.7. Data Analysis

All statistical analysis were performed with Statistica (SAS, Cary, NC, USA). Differences in the expression levels of CsTIFY in different organs and differences time points treated with *C. camelliae* infection were analyzed by one-way ANOVA (Tukey’s multiple comparison test. The expression levels of CsTIFY in different treatments (JA, mechanical wounding and attack of tea geometriod) between treatment and control were analyzed for significance by Student’s *t*-test.

## Figures and Tables

**Table 1:** Basic information of CsTIFY gene family.

Gene ID	Gene Name	CDS Length (bp)	Protein Length (aa)	TIFY Motif
CSS0010510	CsJAZ1	702	233	TIFYGG
CSS0031905	CsJAZ2	885	294	TIFYAG
CSS0022053	CsJAZ3	1197	398	TIFYAG
CSS0012514	CsJAZ4	1197	398	TIFYAG
CSS0028188	CsJAZ7	411	136	TIFYNG
CSS0034430	CsJAZ8	654	217	TIFYNG
CSS0011019	CsJAZ10	786	361	TIFYGG
CSS0031620	CsPPD	1008	335	TIFYCG
CSS0050449	CsZML7	912	303	TLSFQG
CSS0037100	CsZML8	912	303	TLSFQG
CSS0003047	CsZML1	846	281	TLSFRG
CSS0017505	CsZML2	972	323	TLSFQG
CSS0004261	CsZML3	978	325	TLSFQG
CSS0050427	CsZML4	1092	363	TLAFEG
CSS0045061	CsZML5	1089	362	TSELSI
CSS0016802	CsZML6	1131	376	TLAFEG

## Supplementary Materials

Supplementary materials can be found at https://www.mdpi.com/1422-0067/21/21/8316/s1. Figure S1, Phylogenetic tree of JAZ proteins from tea plant and Arabidopsis. The predicted full-length amino acid sequences of seven CsJAZ and 12 AtJAZ were used to construct a phylogenetic tree using MEGA5.1 by the neighbor-joining method; Table S1, Primers for qRT-PCR analysis.
