# Supplementary material for: Genome-Wide Identification of the Tify Gene Family and Their Expression Profiles in Response to Biotic and Abiotic Stresses in Tea Plants (Camellia sinensis)
Source: Int J Mol Sci. 2020 Nov 5;21(21):8316. doi: 10.3390/ijms21218316 (PMC7664218; doi:10.3390/ijms21218316)
Supplement: Supplementary file 1 [file ijms-21-08316-s001.pdf]

Supplementary Table S1. Primers for qRT-PCR analysis.

Gene	Primers for Real-time PCR	
	Forward Primer (5'–3')	Reverse Primer (5'–3')
CsJAZ1	TAAAACCGCCCCAATGACGA	TTCCGAGGGCTGGAATAGGA
CsJAZ2	TCGTCGAGCTTCTCTCAGAC	CGTCACCGGAGTTCCATTTC
CsJAZ3	TTGCTTCTGTTGCAGGAACC	GGACCTGAACCTGAGCTCTT
CsJAZ4	TGGCCAACCAAACAAACCAA	CTCGTGGGAACGACTGAAAC
CsJAZ7	CGGATTACAACCACCACCAC	AAATCGTTAGCGGCTGTTCC
CsJAZ8	GCTAACTTTCGTGTCGCCTT	CCTCTGTCCGGATGTGTTCT
CsJAZ10	CCGCAGGGCATTAAAGTCTC	GTGAAGGGAAAGGTGAGGGA
CsPPD	TTCGTGAAGACAACCCTGCAGTT	GCACCGGGTAGGAGTGTTG
CsZML1	CGTACCGACTGTTCCAACCA	TGGCCCTTCTTACGTTGCAT
CsZML2	CGGTGCTGGCAGCGT	GAAGCAGCCCTTTGTGGTTG
CsZML3	GCCGATCAGCTAACCCTCTC	GAAGCAGCCCTTTGTGGTTG
CsZML4	ACGTGACTTCCGTGAATGCT	GGTATTTCACGCCCTCCCAA
CsZML5	TGAAGACGGAGGAGGAGGAG	TGCAAAGAGTTCTCGGACCC
CsZML6	TGTGGGCAAACAAGGGAACT	TTTGGATCTGATGTGGCCGT
CsZML7	ACAAGCATGCCTACTGTCCC	AATTTCCATTCTCATTCTGGCTCA
CsZML8	TGAGCCAGAATGAGAATGGAAAT	ATCACTGCCAGTTCCAGCAA
CsGAPDH	TTTTTGGCCTTAGGAACCCAGAGG	GGGCAGCAGCCTTATCCTTATCAGT

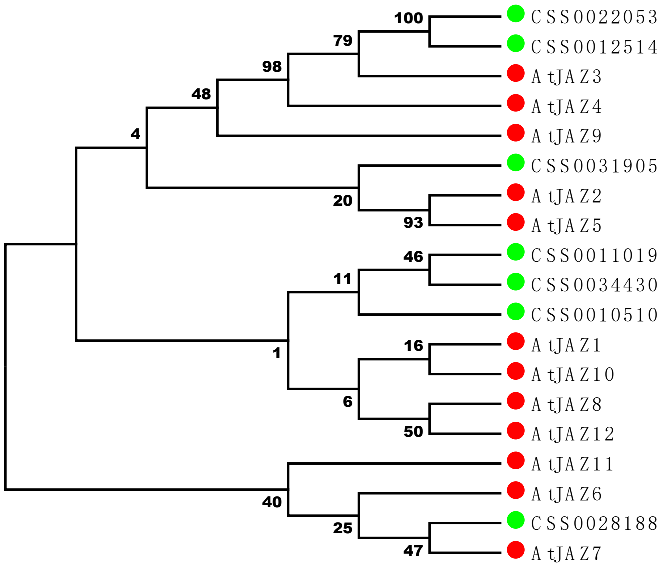

Supplementary Figure S1. Phylogenetic tree of JAZ proteins from tea plant and *Arabidopsis*. The predicted full-length amino acid sequences of seven CsJAZ and 12 AtJAZ were used to construct a phylogenetic tree using MEGA5.1 by the neighbor-joining method.
